# Electronic Health Literacy in Swiss-German Parents: Cross-Sectional Study of eHealth Literacy Scale Unidimensionality

**DOI:** 10.2196/14492

**Published:** 2020-03-13

**Authors:** Sibylle Juvalta, Matthew J Kerry, Rebecca Jaks, Isabel Baumann, Julia Dratva

**Affiliations:** 1 Institute of Health Sciences Department of Health Zurich University of Applied Sciences Winterthur Switzerland; 2 University of Basel Basel Switzerland

**Keywords:** health literacy, eHealth, eHEALS, unidimensionality, multidimensionality, factor analysis, item response theory (IRT), bifactor model, validity

## Abstract

**Background:**

Parents often use digital media to search for information related to their children’s health. As the quantity and quality of digital sources meant specifically for parents expand, parents’ digital health literacy is increasingly important to process the information they retrieve. One of the earliest developed and widely used instruments to assess digital health literacy is the self-reported eHealth Literacy Scale (eHEALS). However, the eHEALS has not been psychometrically validated in a sample of parents. Given the inconsistency of the eHEALS underlying factor structure across previous reports, it is particularly important for validation to occur.

**Objective:**

This study aimed to determine the factor structure of the German eHEALS measure in a sample of parents by adopting classic and modern psychometric approaches. In particular, this study sought to identify the eHEALS validity as a unidimensional index as well as the viability for potential subscales.

**Methods:**

A cross-sectional design was used across two purposive sampling frames: online and paper administrations. Responses were collected between January 2018 and May 2018 from 703 Swiss-German parents. In addition to determining the sampling characteristics, we conducted exploratory factor analysis of the eHEALS by considering its ordinal structure using polychoric correlations. This analysis was performed separately for online–based and paper–based responses to examine the general factor strength of the eHEALS as a unidimensional index. Furthermore, item response theory (IRT) analyses were conducted by fitting eHEALS to a bifactor model to further inspect its unidimensionality and subscale viability.

**Results:**

Parents in both samples were predominantly mothers (622/703, 88.5%), highly educated (538/703, 76.9%), of Swiss nationality (489/703, 71.8%), and living with a partner (692/703, 98.4%). Factor analyses of the eHEALS indicated the presence of a strong general factor across both paper and online samples, and the Wilcoxon rank-sum test indicated that the eHEALS total sum score was not significantly different between the paper and online samples (*P*=.12). Finally, the IRT analyses indicated negligible multidimensionality, insufficient subscale reliability after accounting for the eHEALS general factor, and a reduced subset of items that could serve as a unidimensional index of the eHEALS across the paper and online samples.

**Conclusions:**

The German eHEALS evidenced good psychometric properties in a parent-specific study sample. Factor analyses indicated a strong general factor across purposively distinct sample frames (online and paper). IRT analyses validated the eHEALS as a unidimensional index while failing to find support for subscale usage.

## Introduction

Parents increasingly use digital sources when seeking information on their child's health [1-3]. Through their accessibility, digital sources offer the opportunity for parents to feel empowered [4] (eg, to verify information received from health professionals), consider alternative treatment options, and develop communal networks with other families and patients with a common disease or condition. However, because the quality and reliability of information from digital sources vary substantially [5], the information can be overwhelming and cause insecurity or anxiety [6-8]. Therefore, eHealth literacy of parents is critical to maximize the potential benefits of digital media for children’s health. eHealth literacy has been defined as “a set of skills required to effectively engage information technology for health” [9].

Research on the eHealth literacy of parents is lacking. A study by Knapp et al [10] in Florida showed that low-income parents of children with special health needs had high levels of internet use for information purposes. However, half of the study participants had difficulties separating high-quality from low-quality information and were not confident using the internet. Similar findings were found in a study of parents whose children had life-threatening illnesses [11]. Both studies used the eHealth Literacy Scale (eHEALS) [9]. Both studies are also older, considering the fast-evolving context of internet acculturation. The eHEALS was developed by Norman and Skinner [12], who defined eHealth literacy as “the ability to seek, find, understand, and appraise health information from electronic sources and apply the knowledge gained to addressing or solving a health problem.” In this regard, the eHEALS pertains to the critical consumption of extant internet content rather than the creation of new content. The eHEALS is a widely applied instrument that has been validated internationally in diverse languages [13]. Adequate internal consistency has been found [13]. However, van der Vaart et al [14] reported weak correlations between the eHEALS scores and tasks on an eHealth performance test. In contrast, more recent research found significant associations between perceptions and performance [15]. In addition, inconsistent results for the factor structure of the eHEALS have been reported. Norman and Skinner [9] proposed a one-factor structure for the original English eHEALS. For the German eHEALS, Soellner et al [16] determined a two-factor structure using confirmatory factor analysis. For an Italian version of the eHEALS applied in the Italian-speaking area of Switzerland, the researchers [17] recommended using the eHEALS total sum score after applying Rasch (item response theory [IRT]) modeling. However, a study with patients at risk of cardiovascular disease in Australia also applied Rasch modeling and concluded that the eHEALS captures different aspects of eHealth literacy, which may have to be scored separately [18]. Neter et al [19] reported a different two-factor solution for adults in Israel aged at least 21 years than the two-factor solution used by Soellner et al [16]. Moreover, two recent studies developed a three-factor solution for the English eHEALS: awareness, skills, and evaluation [20,21]. A three-factor solution was also reported in an IRT analysis of eHEALS, although the authors noted that substantially high interfactor correlations “support an overarching structure of eHEALS” [22]. Notably, both studies implementing IRT analyses of the eHEALS found measurement properties (item difficulties) reflective of their study samples. That is, Diviani et al [17] found wide variability in item difficulties in a broad sample of people aged 16-71 years, and Stellefson et al [22] found high item difficulties in a sample narrowed to older adults.

The eHEALS was originally constructed for broad usage, as creators Norman and Skinner [9] state, “this article describes the development and psychometric evaluation of a measure of eHealth literacy designed for broad use in supporting consumer eHealth in public health and clinical care” (p2). Subsequent research has reported unstable latent factor structures underlying the eHEALS measure, despite the conventional understanding that “broader constructs are stabilized with broad factors” [23]. Given the inconsistent results for the eHEALS, we aimed to establish the psychometric structure of the eHEALS in parents participating in the Digital Parental Counselors study (in German: Digitale Elternratgeber), which investigated digital media use by parents for their children’s health in the German-speaking area of Switzerland [24]. Specifically, we aimed to explore the factor structure of the German eHEALS and to assess the viability of subscales using IRT and bifactor modeling. We also addressed the methodological issues concerning the handling of Likert scales and the use of factor analysis experienced with previous research of the eHEALS.

## Methods

### Study Population

The study population consisted of a population-based sample of parents with children aged 1-24 months. The birth registries of Zürich and 5 municipalities in the canton of Zürich, which were selected using convenient sampling, provided randomly selected names and addresses of 2573 mothers who gave birth in the previous 24 months. Urban and rural municipalities were included to represent the urban/rural distribution in the German part of Switzerland (75%/25%). The ethical commission of the Canton of Zurich, based on the Swiss Federal Act on Research involving Human Beings, exempted the study from ethics review (BASEC Req-2017-00817).

### Data Collection

The data were collected between January 2018 and May 2018. To increase the response rate, we applied a mixed-mode approach using online and paper versions of the questionnaire. The questionnaire consisted of three main parts: (1) sociodemographic characteristics of the parent and child, (2) digital media use in relation to the child’s health, and (3) health-related variables and eHealth literacy.

Parents received a postal invitation letter with a link to the online questionnaire. After the first postal reminder, parents received a paper questionnaire with the second and last reminder letters.

### eHealth Literacy Scale

The eHEALS consists of 8 items (see Table 1). Responses are provided using a 5-point Likert scale ranging from 1 (strongly disagree) to 5 (strongly agree), with total scores ranging from 8 to 40 points. Higher scores reflect higher eHealth literacy. The eHEALS was developed based on the concept that eHealth literacy is composed of core skills grouped into analytical skills such as media literacy and context-specific skills such as health literacy [12]. The eHEALS does not measure these skills directly, but rather “the consumer's perceived skills and comfort with eHealth” [9]. For this study, we used the German eHEALS version developed by Soellner et al [16], who translated and cross-validated the original English version by Norman and Skinner [9] in a German sample.

**Table 1:** Example of past studies exploring the latent structure of the eHealth Literacy Scale (eHEALS) measure.

Variables			Studies		
			Norman & Skinner (2006) [9]		Soellner et al (2014) [16]
**Sample characteristics**					
	Population	Canadian students		German students	
	Sample size	664		327	
	Age (years), range	13-21		16-21	
**Factor Solution**					
	Construction	Original		German translation, as reported here	
	Structure	1-factor		2-factor	
**Factors**					
	eHEALS item 1	I know how to find helpful health resources on the Internet		Ich weiss, wie ich im Internet nützliche Gesundheitsinformationen finde^a^	
	eHEALS item 2	I know how to use the Internet to answer my questions about health		Ich weiss, wie ich das Internet nutzen kann, um Antworten auf meine Fragen rund um das Thema Gesundheit zu bekommen^a^	
	eHEALS item 3	I know what health resources are available on the Internet		Ich weiss, welche Quellen für Gesundheitsinformationen im Internet verfügbar sind^a^	
	eHEALS item 4	I know where to find helpful health resources on the Internet		Ich weiss, wo im Internet ich nützliche Gesundheitsinformationen finden kann^a^	
	eHEALS item 5	I know how to use the health information I find on the Internet to help me		Ich weiss, wie ich Informationen aus dem Internet so nutzen kann, dass sie mir weiterhelfen^a^	
	eHEALS item 6	I have the skills I need to evaluate the health resources I find on the Internet		Ich bin in der Lage, Informationen, die ich im Internet finde, kritisch zu bewerten^b^	
	eHEALS item 7	I can tell high quality health resources from low quality health resources on the Internet		Ich kann im Internet zuverlässige von Fragwürdigen Informationen unterscheiden^b^	
	eHEALS item 8	I feel confident in using information from the Internet to make health decisions.		Wenn ich gesundheitsbezogene Entscheidungen auf Basis von Informationen aus dem Internet treffe, fühle ich mich dabei sicher^a^	

^a^Information seeking.

^b^Information appraisal.

### Data Analysis

We performed three different analyses to answer three distinct questions. The first analyses were descriptive and concerned differences in the sample characteristics. The second analyses were based on classical test theory and concerned the general factor strength for each sampling frame (online vs paper). The third analyses involved modern IRT and concerned unidimensionality assumptions and item-level bias across the online and paper administration samples.

#### Descriptive Analysis

Frequencies of the sociodemographic characteristics of the responding parents and their children were analyzed. Separately for the paper, online, and total samples, the single item and total sum eHEALS scores are reported as the median, skew, and mean. The total sum scores from the online and paper questionnaires were compared using the non-parametric Wilcoxon rank-sum test [25] prior to merging the data. All descriptive analyses were computed with Stata 15.0 (StataCorp LLC, College Station, TX).

#### Classical Test Theory Analysis

For Likert scales, it is recommended to consider their ordinal structure for factor analysis [26]. As the conventional exploratory factor analysis (EFA) treats variables in a metric manner, polychoric correlations were computed to take into account the ordinal structure of the eHEALS items (see Multimedia Appendix 1).

The detailed results of the EFA conducted with the psych package [27] in R (R Foundation for Statistical Computing, Vienna, Austria) are displayed in Multimedia Appendix 1. Furthermore, a series of parallel analyses were conducted to determine the single-factor strength of the eHEALS measure across the sampling frames [28] using SPSS version 25.0 (IBM Corp, Armonk, NY). Finally, the internal consistency of the eHEALS scale was assessed using the McDonald’s omega coefficient [29].

#### Item Response Theory Analysis

**Figure 1:**
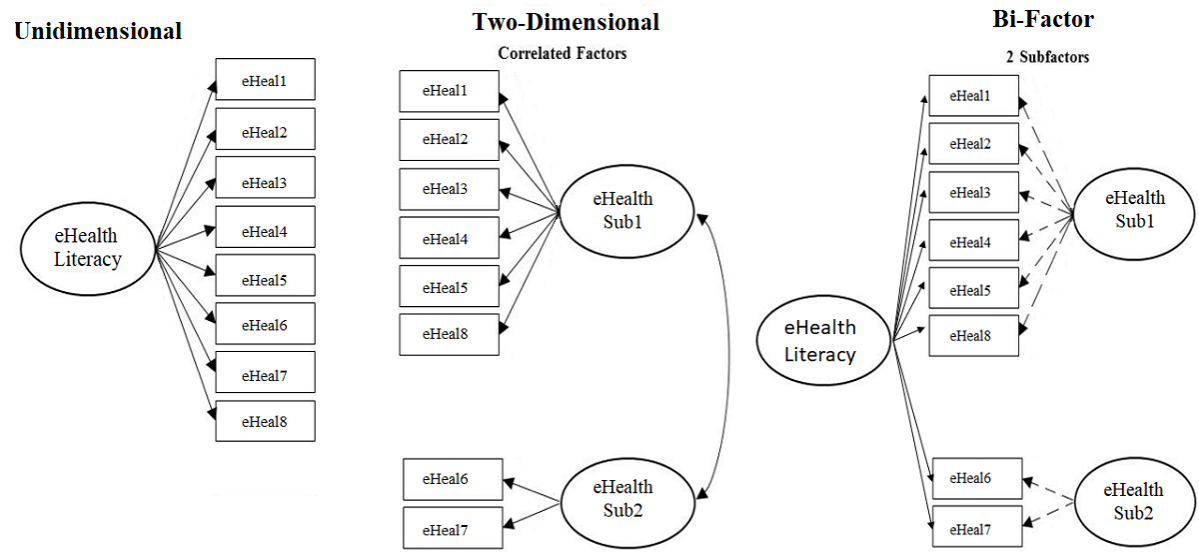
Graphical representations of the eHealth unidimensional, correlated two-dimensional, and bi-factor two-dimensional models. The solid lines in the bi-factor model indicate unidimensional primacy over the residualized sub-dimensions (hashed arrows). eHealth: electronic health; eHeal: eHealth Literacy.

Previous findings have indicated unstable latent structures (number of eHEALS factors). In such a situation, the bifactor model helps to determine how useful it is to form subscales and examine if unidimensional IRT models can be fit to such multidimensional data [30]. We therefore inspected the eHEALS using a bifactor model. In a bifactor model, a general factor is generated through all test items. Additionally, group factors are established out of the residual variance shared by subsets of items [31] (see Figure 1). In addition to the bifactor model, further IRT understanding based on the internal psychometric structure of the eHEALS was specifically examined in terms of general factor strength (appropriate unidimensional scores) and substantive multidimensionality (item bias). IRTPRO v 4.2 (Scientific Software International, Skokie, IL) was used to run the IRT analyses, and the unidimensional indices were computed using the Bifactor Indices Calculator [32], including McDonald´s omega [29].

## Results

### Descriptive Analysis

A total of 842 parents or caretakers responded to the survey, and we excluded 73 responses during the data cleaning process for the following reasons: incomplete questionnaire (n=31), missing answers to key questions on parental digital health information seeking (not including the eHEALS items; n=40), non-plausibility of key questions (n=1), and duplicate entry (n=1). This resulted in 769 observations corresponding to a response rate of 30% for the overall study. The online questionnaire was completed by 429 participants (429/769, 56%), and 340 participants (340/769, 44%) completed the paper questionnaire. For the analysis of the eHEALS, 67 additional observations had to be discarded because 52 had missing values for all eHEALS items and 15 had missing values for single eHEALS items.

This led to a final online sample of 388 participants and a final paper sample of 315 participants. Table 2 provides the summary descriptive statistics of the whole sample (N=703). Of the sample, 88.5% (622/703) of the participants were mothers, 76.9% (538/703) reported a university degree or higher vocational education as their highest educational level, and 45.4% (294/703) earned a monthly income >9000 Swiss Francs (US $9080; €8020). The majority (489/703, 71.8%) were Swiss, and almost all study participants (692/703, 98.4%) indicated they lived with a partner.

**Table 2:** Summary of the sample characteristics, N=703.

Characteristic			Participants, n (%)	
**Parental sex**				
	Mother			622 (88.5)
	Father			78 (11.1)
	Other			3 (0.4)
Age (years)			35.7 (4.3)^a^	
**Education level**				
		Lower education	162 (23.1)	
		Higher education	538 (76.9)	
**Nationality**				
		Swiss	489 (71.8)	
		Other	192 (28.2)	
**Living with a partner**				
		Yes	692 (98.4)	
		No	11 (1.6)	
**Household net monthly income (CHF)**				
		<4500	27 (4.2)	
		4500-6000	94 (14.5)	
		6000-9000	233 (36.0)	
		>9000	294 (45.4)	
**Child’s sex**				
		Female	349 (49.9)	
		Male	350 (50.1)	
Child’s age (months)			14.8 (7.1)^a^	
**First child**				
		Yes	353 (51.2)	
		No	337 (48.8)	
Digital media use score^b^			7.88 (4.13)^a^	
eHEALS total sum score			29.0 (5.9)^a^	

^a^Mean (SD).

^b^Sum score on how often parents use several digital media for general child health and development (ranging from 0-24).

Concerning the eHEALS items, there were no differences in the individual item responses between the paper and online modes, except for item 3, where the online sample yielded a lower median. The distributions for all items for both the paper and online samples were slightly negatively skewed. As there was no significant difference in the eHEALS total sum scores between the online and paper samples (*P*=.12), the analysis was conducted using the total sample (see Table 3).

**Table 3:** Descriptive statistics of the individual eHealth Literacy Scale (eHEALS) items and total sum score for the online, paper, and total samples.

eHEALS item	Online (n=388)			Paper (n=315)			Total sample (n=703)		
	Median	Skew	Median		Skew	Median		Mean (SD)	*P* value
Item 1	4	–1.02	4		–0.76	4		3.7 (1.1)	N/A
Item 2	4	–0.91	4		–0.95	4		3.7 (1.0)	N/A
Item 3	3	–0.55	4		–0.55	4		3.4 (1.0)	N/A
Item 4	4	–0.57	4		–0.61	4		3.5 (1.0)	N/A
Item 5	4	–0.87	4		–0.95	4		3.7 (0.9)	N/A
Item 6	4	–1.40	4		–1.23	4		4.2 (0.9)	N/A
Item 7	4	–0.77	4		–0.81	4		3.9 (0.9)	N/A
Item 8	3	–0.24	3		–0.21	3		3.0 (1.1)	N/A
eHEALS total sum score	30	–0.67	29		–0.76	30		28.5 (6.2)	0.12^a^ (0.12^b^)

^a^Median test.

^b^Wilcoxon rank-sum test.

### Classical Test Theory Analysis

**Figure 2:**
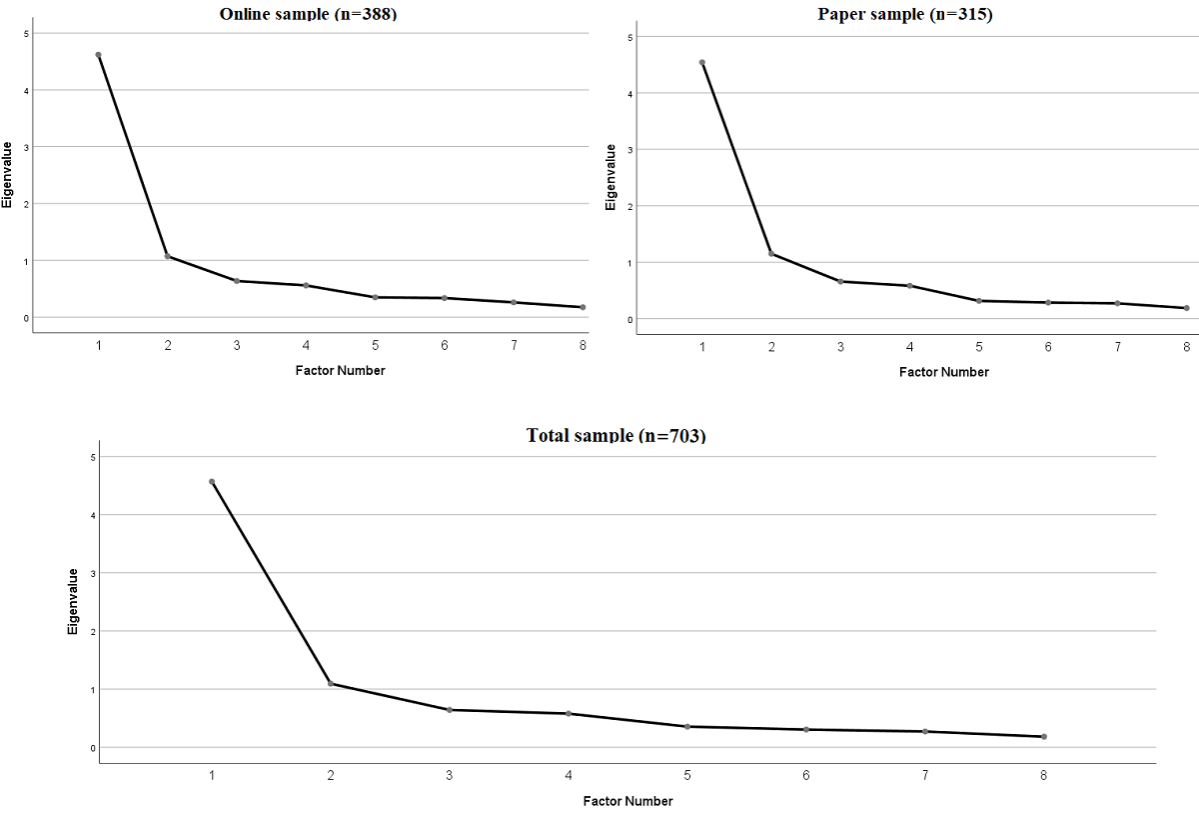
Exploratory factor scree plots to examine the strength of the general factors across the online, paper, and total samples.

Given the non-significant difference in the eHEALS total sum scores between theoretically distinct sampling frames (paper and online collection methods), a series of exploratory factor analyses were conducted to examine the strength of the general factor across the samples. As shown in Figure 2, the first eigenvalues indicated the presence of a strong general factor across sampling frames as well as for the total combined sample. Specifically, the first and second eigenvalue ratios across the samples were 4.62 and 1.07 = 4.32 (online), 4.54 and 1.15 = 3.95 (paper), and 4.57 and 1.09 = 4.19 (total). The large eigenvalue ratios suggest negligible multidimensionality across sample frames [33]. This was supported by omega reliability estimates across the samples of 0.90 (online), 0.89 (paper), and 0.89 (total) [29].

When considered collectively with the non-significant difference in eHEALS total scores between sampling frames, we used IRT to test the unidimensionality assumptions with the total eHEALS sample.

### Item Response Theory Analysis

Separate bifactor models with two group factors and three group factors were computed. A direct model comparison between the bifactors indicated the two-group factor model exhibited significantly greater fit than the three-group factor model (χ^2^_1_=58.4, *P*<.001).

Furthermore, the item loadings between the unidimensional and bifactor models were compared to determine the impact on the bias from ignoring suspected multidimensionality (literally, comparing across models with and without additional dimensions). Table 4 lists the loadings across the eHEALS unidimensional and bifactor models.

The average relative parameter bias (0.09; Table 5) value indicates that the impact of ignoring the multidimensionality of eHEALS by using unidimensional scores was negligible [34].

**Table 4:** Item response theory electronic health item loadings, N=703.

Item	Unidimensional model	Bifactor model with two group factors		
		General Factor	Factor 1	Factor 2
1	0.81	0.87	–0.09	N/A
2	0.88	0.94	–0.07	N/A
3	0.89	0.81	0.40	N/A
4	0.91	0.82	0.56	N/A
5	0.88	0.85	0.11	N/A
6	0.57	0.49	N/A	0.74
7	0.68	0.6	N/A	0.68
8	0.66	0.63	0.10	N/A

**Table 5:** Item response theory electronic health unidimensionality indices, N=703.

Unidimensionality index	Value
ECV^a^	0.76
Omega reliability	0.99
Hierarchical omega	0.92
H replicability	0.95
Factor determinacy	0.99
ARPB^b^	0.09
IECV^c^ (number of items >0.80)	5

^a^ECV: estimated common variance.

^b^ARPB: average relative parameter bias.

^c^IECV: item estimated common variance.

To verify this inference, first, we examined the correlation between the eHEALS and a meaningful substantive variable from the survey with the parents (sum score on how often parents use several digital media for general child health and development), which was significant in the expected direction (r=0.29, *P*<.01). Second, we examined the difference in this correlation between the unidimensional and bifactor scores of the eHEALS and digital media use for general child health and development. The results indicated observably small changes in the correlation (r_Δ_=0.02), which was tested and was not significant (z_(1)_=0.37, *P*=.71) for the eHEALS total sum score correlation with this substantive variable.

## Discussion

The findings of this study support the usefulness of the eHEALS measure as a unidimensional index for further studies. Specifically, we found a strong general factor of the eHEALS across distinct sampling frames as well as adequate reliability. Furthermore, the IRT analyses indicated minimal distortion of the primary factor from ignoring potential multidimensionality, and subscale reliabilities were inadequate to recommend further usage.

With respect to the EFA, this study used a different methodology to add to the current discussion of the eHEALS factor structure. Norman and Skinner [9] and other researchers factorizing the eHEALS [17,35-37] used principal component analysis for data reduction, while we implemented factor analysis to identify underlying latent constructs [38]. Another issue with previous analyses of the eHEALS is that the Likert scales were treated as continuous variables. In our analyses, we considered the ordinal structure of the eHEALS items using a polychoric correlation matrix when performing the EFA. Our results show the predominance of a single factor across both paper and online samples. Importantly, we also found no significant difference in the eHEALS total sum scores between the paper and online sampling frames. Unidimensionality assumptions were further tested using IRT analyses.

Given the construction and theorized application of eHEALS as a broad construct, our IRT analyses included a bifactor model. Consistent with previous IRT analyses, our findings indicated unidimensionality of the eHEALS [9,17,39]. Our findings also agree with the sampling-measurement interrelationship, such that our difficulty parameters were lower in our relatively young sample than the average. Further evidence from the comparison of the bifactor modeling of the primary factor suggested that the distortion from ignoring the potential multidimensionality in our multimodal sample was negligible. Furthermore, the empirical reliability after accounting for the general factor was inadequate to support future subscale use. Our results are not directly comparable to those from the original study by Soellner et al [16] in a German sample, since their results were not based on the IRT methodology. There is some agreement between the findings, given that two group factors were a better fit for the bifactor model than three group factors. These findings suggest two future applications for the German eHEALS. First, researchers may wish to pilot new items to expand subscales to achieve sufficient reliability. Second, researchers may wish to use a subset of items for purely unidimensional purposes. In the latter case, we refer readers to a potential core subset that could be comprised of the 5 items in Table 4 with item estimated common variances >.80. These are reported in Multimedia Appendix 2 with a preliminary differential item functioning analysis that indicated no bias of the items across paper and online samples.

In summary, the results support the broad but unidimensional factor structure of the German eHEALS. Our ordinal factor analysis supports the presence of a strong general factor. Furthermore, the item response theory analysis using bifactor models with one general factor and two or three group factors showed that the model with two group factors fitted better than the one with three factors. Comparing this bifactor model with two group factors with the unidimensional loadings did not suggest a substantial difference in primary loadings. Finally, we found no support for using eHEALS subscales.

The use of subscales in previous research [16,20,40] may underpin the recommendation to have 3 to 5 items per common factor. Although an advantage of the eHEALS might be its short length (only 8 items) from a methodological viewpoint, the factors may be underdetermined if the items were split into subscales [38]. Still, the benefit of different subdimensions of eHealth literacy would lie in the ability to identify possible areas of intervention. Conceptual models on general health literacy include the components of understanding, appraising, and applying health information [41]. For example, item 8 of the eHEALS “I feel confident in using information from the internet to make health decisions” could reflect the dimension of applying health information. As others have already indicated [22], to add further items to help discriminate components of eHealth literacy, we require a better understanding of the concepts study participants associate with particular eHEALS items. However, given that past findings have indicated a mixed number of factors underlying the eHEALS, researchers should carefully consider the overspecification value relative to the general stability. In this study, the unidimensional bifactor model of the eHEALS was stable across the two distinct samples (online and paper modes).

### Limitations

This study has some limitations. Although the parents were asked about their own eHealth literacy, it is likely that the questions on child health prompted the parents to answer the eHEALS items from the perspective of child health. This would explain the parents’ reluctance to make decisions based on internet-based health information (item 8 of the eHEALS). Therefore, in comparison with studies on adult eHealth literacy, parental eHealth literacy might be lower. However, the high information needs of parents, especially right after birth, might have increased eHealth literacy simply through practice and experience. Regarding the sample characteristics, the generalizability of our findings might be limited by the fairly low response rate (769/842, 30%) and the uniquely high socioeconomic status.

Another limitation is that measurement invariance was not assessed in terms of the participants’ individual characteristics. For example, future studies could focus specifically on the generalizability by gender of our proposed unidimensional eHEALS. This was not the focus of the current study and will be addressed in further analyses. It is important, furthermore, for future researchers to consider the relevance of their samples when studying eHEALS measurement properties. This study aimed to extend the application of the eHEALS among new parents.

### Conclusions

This study suggests that the German eHEALS possesses a broad, unidimensional factor structure among Swiss-German parents. Although the two samples differed with respect to participant characteristics such as age, education, and income, we failed to find a significant difference in the eHEALS total sum scores. The underrepresentation of participants of lower socioeconomic status, not only in our study but also in many other studies on digital health, warrants future studies to over-sample this population. We found similar factor structures and item properties irrespective of application mode. That is, the EFAs suggested a strong general factor. Finally, bifactor modeling did not outperform the unidimensional model, and subscales were unsupported because of low reliability. While using the total sum score is appropriate to assess eHealth literacy, further development and refinement of the eHEALS are proposed to address specific sub-domains of eHealth literacy. For any sample, practitioners should use only the eHEALS total score, and future research aiming to utilize subscales should expand the eHEALS item pool for empirical testing.

## Appendix

Multimedia Appendix 1 Detailed protocol of the exploratory factor analysis with polychoric correlation matrix.

Multimedia Appendix 2 Potential core set of the German eHEALS and differential item functioning analysis (DIF).

## Abbreviations

EFA: exploratory factor analysis.
eHEALS: eHealth Literacy Scale.
IRT: item response theory.
