# Supplementary material for: Electronic Health Literacy in Swiss-German Parents: Cross-Sectional Study of eHealth Literacy Scale Unidimensionality
Source: J Med Internet Res. 2020 Mar 13;22(3):e14492. doi: 10.2196/14492 (PMC7101498; doi:10.2196/14492)
Supplement: Multimedia Appendix 1 [file jmir_v22i3e14492_app1.docx]

Multimedia Appendix 1: Detailed protocol of exploratory factor analysis with polychoric correlation matrix

With on overall value of 0.86, the KMO test suggested a good factorability for the sample.

The parallel analysis (see figure 1) proposed the extraction of three factors. The inspection of the scree plot shows a steep drop of the Eigenvalues between one and two factors which levels off after three factors.

An orthogonal rotation was chosen to maximize the unique effect of the different subfactors. The three-factor solution showed a first factor with high loadings of the items 1 and 2 and a medium loading of item 5, another factor where items 3&4 loaded onto and a third factor composed by items 6&7. This factor solution seems plausible, as it reflects the three factor solution proposed by Sudbury-Riley et al. [1]: "awareness" (items 3&4), "skills "(items 1,2&5) and "evaluate" (items 6&7). However, item 8 fails to load on one of the three factors with a loading higher than 0.5. It loads with 0.38 on the skills factor, with 0.39 on the awareness factor and with 0.31 on the evaluate factor.

The rotation was done again two factors and one factor. Thereby, the two factor model represented the factor structure proposed by Soellner et al. [2]: As shown in table 2, a first factor is characterized by high loadings of the items 1 to 5 and 8 (“information seeking”) and another factor where items 6&7 loaded onto (“information appraisal”). This factor solution is better interpretable, as item 8 only loads to the information appraisal factor.

The three-factor model explains 74% of the variance, the two factor 69% and the one factor 58%. Other model parameters are found in table 3 and show that model fit is suboptimal for all factor solutions. However, global fit indices are not optimal for determining the number of factors [3] and are overfactoring, i.e. the model fit gets better the more factors are added [4].

Table 1: Descriptive statistics of the eHEALS items and polychoric correlation matrix

| Polychoric correlation matrix | | | | | | | |
| --- | --- | --- | --- | --- | --- | --- | --- |
|  | | | | | | | |
| Item 1 | Item 2 | Item 3 | Item 4 | Item 5 | Item 6 | Item 7 | Item 8 |
|  |  |  |  |  |  |  |  |
| 1 |  |  |  |  |  |  |  |
| 0,78 | 1 |  |  |  |  |  |  |
| 0,6 | 0,7 | 1 |  |  |  |  |  |
| 0,63 | 0,72 | 0,84 | 1 |  |  |  |  |
| 0,64 | 0,73 | 0,66 | 0,73 | 1 |  |  |  |
| 0,35 | 0,39 | 0,4 | 0,37 | 0,57 | 1 |  |  |
| 0,42 | 0,48 | 0,5 | 0,51 | 0,61 | 0,74 | 1 |  |
| 0,48 | 0,52 | 0,5 | 0,56 | 0,56 | 0,32 | 0,48 | 1 |

Figure 1: Parallel analysis for the total sample


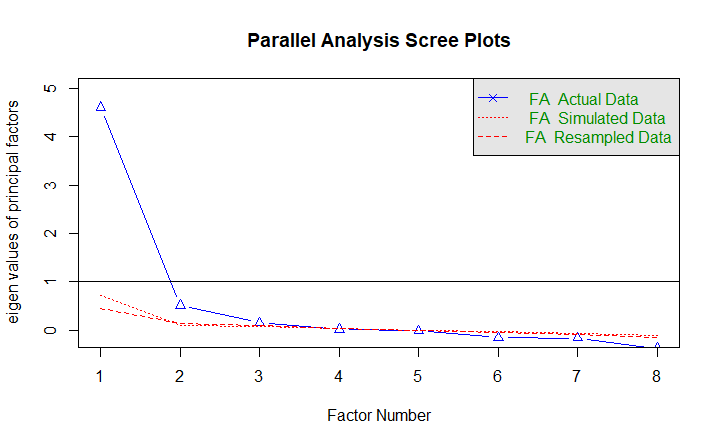


Table 2. Rotated factor loadings for the total sample ^a-b^

|  | Factor 1 | Factor 2 |
| --- | --- | --- |
|  |  |  |
| Item 1 | 0.75 |  |
| Item 2 | 0.84 |  |
| Item 3 | 0.78 |  |
| Item 4 | 0.85 |  |
| Item 5 | 0.71 |  |
| Item 6 |  | 0.83 |
| Item 7 |  | 0.81 |
| Item 8 | 0.55 |  |

^a^ factors rotated with varimax rotation

^b^only loadings >0.5 are displayed

Table 3: Model fit for different factor solutions

| Number of factors | **TLI^a^** | **RMSEA^b^** | **CI RMSEA^c^** | **BIC^d^** |
| --- | --- | --- | --- | --- |
|  |  |  |  |  |
| 3 factors | 0.954 | 0.098 | 0.075-0.121 | 11.31 |
| 2 factors | 0.835 | 0.185 | 0.168-0.202 | 267.59 |
| 1 factor | 0.734 | 0.235 | 0.221-0.248 | 734.02 |

**^a^** TLI: Tucker Lewis Index

**^b^** RMSEA: root mean square error of approximation

**^c^** CI RMSEA: confidence interval of RMSEA

^d^ BIC: Bayesian information criterion

1. Sudbury-Riley L, FitzPatrick M, Schulz PJ. Exploring the Measurement Properties of the eHealth Literacy Scale (eHEALS) Among Baby Boomers: A Multinational Test of Measurement Invariance. Journal of Medical Internet Research 2017;19(2):e53. [doi: 10.2196/jmir.5998]

2. Soellner R, Huber S, Reder M. The concept of eHealth literacy and its measurement: German translation of the eHEALS. Journal of Media Psychology [Internet] 2014;26(1). Available from: https://pub.uni-bielefeld.de/publication/2693761

3. Clark DA, Bowles RP. Model Fit and Item Factor Analysis: Overfactoring, Underfactoring, and a Program to Guide Interpretation. Multivariate Behavioral Research 2018 Jul 4;53(4):544–558. PMID:29683723

4. Kerry MJ, Wang R, Bai J. Assessment of the Readiness for Interprofessional Learning Scale (RIPLS): An item response theory analysis. Journal of Interprofessional Care 2018 Sep 3;32(5):634–637. PMID:29648892
