# Supplementary material for: Electronic Health Literacy in Swiss-German Parents: Cross-Sectional Study of eHealth Literacy Scale Unidimensionality
Source: J Med Internet Res. 2020 Mar 13;22(3):e14492. doi: 10.2196/14492 (PMC7101498; doi:10.2196/14492)
Supplement: Multimedia Appendix 2 [file jmir_v22i3e14492_app2.docx]

Multimedia Appendix 2: Potential core set of the German eHEALS and differential-item functioning analysis (DIF)

Preliminary DIF analyses indicated no significant item bias across online and paper samples (see, table below). The total test-characteristics score for the core 5-item eHEALS across online and paper samples is displayed in the figure below. Finally, for researchers interested in implementing a unidimensional version of the eHEALS exclusively in either an online or paper sample may refer to prior population parameter estimates for each sample reported in the right-hand column of the table.

| Item numbers, DIF tests, and construct loading estimates | | | | | | | |
| --- | --- | --- | --- | --- | --- | --- | --- |
| Online | Paper | Total *X*^2^ | *d.f.* | *P* |  | *λ*_1_ | *s.e.* |
| 1 | 1 | 13.7 | 5 | 0.02 |  | .90 / .78 | .03 / .05 |
| 2 | 2 | 5.6 | 5 | 0.35 |  | .95 / .89 | .02 / .04 |
| 3 | 3 | 3.7 | 5 | 0.59 |  | .79 / .81 | .05 / .04 |
| 5 | 5 | 4.1 | 5 | 0.54 |  | .87 / .80 | .03 / .05 |
| 8 | 8 | 4 | 5 | 0.55 |  | .64 / .61 | .06 / .07 |
